# Prevalence of pemphigus in Colombia from 2013 to 2017 according to data from the National Health Registry^⋆^

**DOI:** 10.1016/j.abd.2021.01.005

**Published:** 2022-05-26

**Authors:** Daniel Fernández-Avila, Laura Charry-Anzola, Lina González-Cardona

**Affiliations:** a Department of Internal Medicine, Hospital Universitario San Ignacio, Pontificia Universidad Javeriana, Bogotá, Colombia; b Medical School, Pontificia Universidad Javeriana, Bogotá, Colombia; c Dermatology Unit, Hospital Universitario San Ignacio, Pontificia Universidad Javeriana, Bogotá, Colombia

Dear Editor,

This is a cross-sectional descriptive study, using the databases of the Comprehensive Social Protection Information System (SISPRO in Spanish) of the Colombian Ministry of Health; the aim is to evaluate the prevalence of pemphigus, its distribution by age and its demographic characteristics in Colombia.

The diagnoses were selected using the codes of the International Classification of Diseases (ICD-10), for Pemphigus Vulgaris (PV) L100, Pemphigus Vegetans (PVE) L101, Pemphigus Foliaceus (PF) L102, Brazilian Pemphigus (fogo selvagem) (BP) L103, Pemphigus Erythematosus (PE) L104, Drug-induced Pemphigus (DP) L105, Other Pemphigus Variants (OPV) L108, and Unspecified Pemphigus (UP) L109 in the period from January 1, 2013 to December 31, 2017. The SISPRO databases, which are feed by the Individual Health Services Delivery Registry, include information on the diagnoses made by physicians in each inpatient or outpatient care in Colombia.^1^ The prevalence was calculated from the last national census conducted in 2005 by the National Administrative Department of Statistics (DANE), with an estimated population in Colombia of 49,291,609 people by 2017.

Between 2013 and 2017, 2,632 confirmed cases of pemphigus were recorded in Colombia, with a prevalence of 5.3 per 100,000 inhabitants. The clinical variants are distributed: PV 31.4%, UP 30.9%, OPV 14.8%, PF 8.6%, PE 7.1%, PVE 3.7%, BP 2.6% and DP 0.5%. The female/male ratio was 1.37:1, which represents 58% for women with 1,524 cases and 42% for men with 1,108 cases (Table 1).

**Table 1:** Patients diagnosed with pemphigus in Colombia between 2013 and 2017.

ICD-10 Diagnosis	Total cases	Cases per 100,0000 inhabitants	Female:Male ratio
Pemphigus Vulgaris	827	1.66	1.3:1
Pemphigus Vegetans	98	0.20	1.7:1
Pemphigus foliaceus	228	0.46	1.2:1
Brazilian pemphigus	69	0.14	2:1
Pemphigus erythematosus	189	0.38	1.1:1
Drug-induced pemphigus	15	0.03	2:1
Other pemphigus variants	391	0.78	1.32:1
Pemphigus, unspecified	815	1.63	1.42:1

Total pemphigus cases: 2,632.

Cases per 100,000 inhabitants: 5.3.

For PV, adults aged 80 years and over had the highest prevalence, with 7 cases per 100,000 inhabitants, for PF adults 75‒79 years of age had the highest prevalence with 2.5 cases per 100,000 inhabitants. In relation to BP adults of 35‒39 years had the highest prevalence with 0.24 cases per 100,000 inhabitants (Fig. 1).

**Figure 1:**
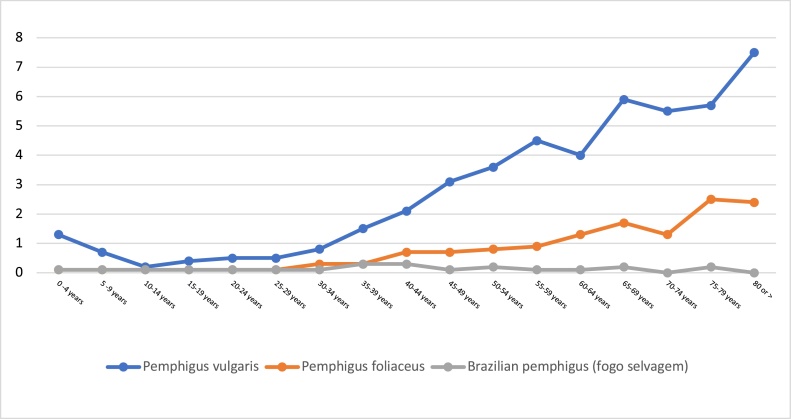
Prevalence of pemphigus vulgaris, pemphigus foliaceus and Brazilian pemphigus (*fogo selvagem*) by age group in Colombia between 2013 and 2017. *Prevalence per 100,000 inhabitants.

The region with the highest prevalence of PV was Chocó, with 4.3 cases per 100,000 inhabitants, for PF was Vaupés, with 13 cases per 100,000 inhabitants, and for BP was Casanare, with 3 cases per 100,000 inhabitants (Table 2).

**Table 2:** Prevalence of Pemphigus Foliaceus (PF), Pemphigus Vulgaris (PV), and Brazilian Pemphigus (BP) by region in Colombia between 2013 and 2017.

Department	Prevalence of PF	Prevalence of PV	Prevalence of BP
Antioquia	0.83	1.75	0.10
Atlántico	0.15	1.46	0.03
Bogotá, D.C.	0.27	1.63	0.04
Bolívar	0.04	1.39	0.13
Boyacá	0.39	1.32	0
Caldas	0.50	3.02	0.20
Caquetá	0.61	1.22	0.20
Cauca	0.92	1.42	0.07
Cesar	0.09	3.22	0
Córdoba	0.51	1.19	0.56
Cundinamarca	0.21	0.90	0.18
Chocó	0.98	4.31	0.19
Huila	0.84	1.01	0.08
La Guajira	0	0.29	0.09
Magdalena	0.38	2.72	0
Meta	0.30	1.30	0.10
Nariño	0.50	2.74	0.16
Norte de Santander	0.21	1.08	0.07
Quindío	0.17	2.27	0.17
Risaralda	0.51	2.59	0.20
Santander	0.19	1.77	0.04
Sucre	0.34	1.49	0.11
Tolima	0.14	1.76	0
Valle del Cauca	0.89	1.80	0.10
Arauca	0	1.49	0.37
Casanare	0.81	2.43	3.79
Putumayo	0.28	1.69	0
San Andrés & Providencia	0	0	0
Amazonas	1.28	0	0
Guainía	7.01	2.33	0
Guaviare	0.87	0	0.87
Vaupés	13.4	0	2.24
Vichada	0	0	0

The prevalence of pemphigus in Colombia is 5.3 per 100,000 inhabitants; this prevalence is almost equal to that reported in the United States, with 5.2 cases per 100,000 inhabitants^2^ and higher than that reported in Latin America, where only one prevalence study was found in Brazil, reporting 3.4 cases per 100,000 inhabitants.^3^ Studies in other continents have a lower prevalence, in Sofia, Bulgaria, a prevalence of 0.38 cases per 100,000 inhabitants was reported,^4^ also in a hospital in southern Saudi Arabia, data on pemphigus were collected, showing a prevalence of 1.56 per 100,000 inhabitants.^5^

The only study showing a higher prevalence was conducted in Iran in 2005, which reported 30 cases per 100,000 inhabitants (Fig. 2).^4^ Our study showed that pemphigus affects more women than men, with a 1.37:1 ratio, as described in some reports in the literature, where women have a higher frequency of the disease.

**Figure 2:**
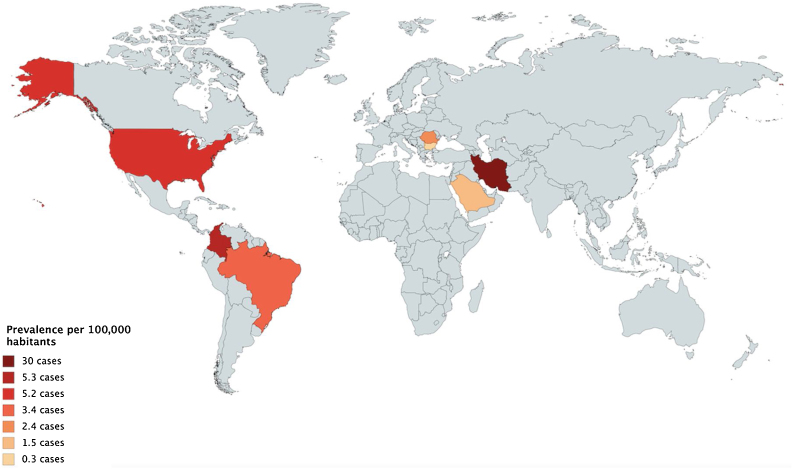
Prevalence of pemphigus worldwide per 100,000 inhabitants.^2^, ^3^, ^4^, ^5^

The highest prevalence of pemphigus was found in the population over 80 years of age, however in PV, variations in age of presentation according to ethnic group have been observed. The earliest age of presentation was in Arabs, compared to Jews, with an average age of 48.6 ± 14 years vs. 57.3 ± 18 years, respectively.^6^

Among the clinical variants of pemphigus, the most frequent was PV accounting for 31.4% and the least frequent was DP with 0.5% of the population. These data are consistent with reported from literature, which shows that PV is the most prevalent with 70%.^5^ Other diagnoses included in ICD-10 are UP with 30.9% and OPV with 14.8% which do not reflect a specific pemphigus variant and do not generate changes when comparing our results with other variants of pemphigus found in the literature.

The highest prevalence of PF was found in Vaupés and Guainía, it is interesting that these two regions are in the southeast of the country, on the border with Brazil; these have extensive rural and jungle areas, and most of their population has a low socioeconomic level. Similar was found in Tunisia, where a higher frequency of PF was reported in the south of the country, a region where people with unfavorable socio-economic conditions live in extensive rural areas.^7^ Likewise, in Peru was found a higher frequency of PF from places with a low socioeconomic level and from Amazon regions.^8^

We present the prevalence, frequency of clinical variants and demographic characteristics of pemphigus in Colombia, the country that so far has the second highest prevalence of this disease worldwide. The limitations of the study are the possible underreporting or wrong reporting by health professionals at the time of entering the diagnosis and the impossibility of calculating the incidence, due to the nature of the data collected.

## Financial support

None declared.

## Authors' contributions

Daniel Fernández-Avila: Approval of the final version of the manuscript; effective participation in research orientation; intellectual participation in propaedeutic and/or therapeutic management of studied cases; manuscript critical review; statistical analysis.

Laura Charry Anzola: Approval of the final version of the manuscript; effective participation in research orientation; intellectual participation in propaedeutic and/or therapeutic management of studied cases; manuscript critical review; statistical analysis.

Lina González Cardona: Critical literature review; data collection, analysis, and interpretation; preparation and writing of the manuscript; study conception and planning.

## Conflicts of interest

None declared.
